# Chronic stress promotes EMT-mediated metastasis through activation of STAT3 signaling pathway by miR-337-3p in breast cancer

**DOI:** 10.1038/s41419-020-02981-1

**Published:** 2020-09-15

**Authors:** Peixin Du, Hao Zeng, Yinan Xiao, Yunuo Zhao, Bo Zheng, Yaotiao Deng, Jie Liu, Boyan Huang, Xinyao Zhang, Keyi Yang, Yu Jiang, Xuelei Ma

**Affiliations:** Department of Medical Oncology, Cancer Center, State Key Laboratory of Biotherapy, West China Hospital, Sichuan University, Sichuan, China

**Keywords:** Breast cancer, Metastasis, miRNAs

## Abstract

Chronic stress could induce cancer metastasis by constant activation of the sympathetic nervous system, while cellular mechanism remains obscure. The aim of this research is to explore the metastasis associated negative effect of chronic stress. The analysis of transcriptome sequencing implied that activation of STAT3 signaling pathway by downregulated miR-337-3p might be a potential mechanism to induce epithelial to mesenchymal transition (EMT) of cancer cell and promote metastasis under chronic stress. We also verified this biological process in further experiments. Downregulation of miR-337-3p could downregulate E-cadherin expression and upregulate vimentin expression in vitro and in vivo. STAT3, related signal pathways of which are involved in metastasis regulation, was directly targeted by miR-337-3p. In conclusion, the above results denoted that activation of miR-337-3p/STAT3 axis might be a potential pathway for the increasing metastasis of breast cancer under chronic stress.

## Introduction

Psychological and emotional problems arise as a result of long-term challenges to physical and mental health and low quality of life in cancer patients^1^. With the development of research in psychological disorders of cancer patients, accumulating evidence showed that psychosocial stress could influence the development, progression, and metastasis of cancer^2,3^. Breast cancer remains the most common cancer in women worldwide and metastasis is widely accepted as the major cause for cancer death^4,5^. In breast tumor bearing mice model, psychosocial stress could induce the formation of pre-metastatic niches in distant organs^6–9^, while blocking stress-associated hormone factors could reduce the metastasis^10,11^. Previous clinical research also indicated that stress-related factors are involved in promoting breast cancer progression^12^. Therefore, a better understanding of underlying mechanism serves to prevent the negative effect of chronic stress on metastasis.

One of the key molecular mechanisms contributing to the metastatic progression is epithelial to mesenchymal transition (EMT), which drives invasion and migration of various cancer including breast cancer^13^. EMT, a conserved cellular program bestowing mesenchymal-like traits on epithelial cells, is often accompanied by changes in expression level of E-cadherin and vimentin^14^. In a clinical research, human breast carcinoma tissues with lower E-cadherin expression could predict poor metastatic-free survival outcome^15^.

miRNAs are short, 18-25 nucleotide-long, noncoding RNA molecules, and they exert impact on cell proliferation, differentiation, and apoptosis^16^. miRNAs could suppress gene expression at both transcriptional and post-transcriptional level by binding to complementary sequences of its target mRNA^17^. miRNAs serve as a crucial components in epigenetic modification for many important biological pathways including EMT^18^. Previous research has suggested that miR-337-3p could regulate the EMT process during cancer progression and metastasis^19^. Several signaling pathway including STAT3 pathway has been reported to participate in the development of EMT process in breast cancer^20^. Previous studies revealed the negative targeting relationship between miR-337-3p and JAK2/STAT3 and further demonstrated that STAT3 is directly targeted by miR-337-3p^21,22^. Additionally, accumulating evidence has validated that miRNAs expression is often dysregulated by stress in cancer, such as chronic stress^23^, heat stress^24^, and surgical stress^25^. The rapid development of sequencing technology enables the detection of genetic changes in mutated cells, which promote the understanding for cancer biology^26,27^. In comparison to the normal control, chronic stress may lead to 20 upregulated miRNAs and 35 downregulated miRNAs detected by using miRNA microarrays method^28^.

In this study, the next-generation sequencing analysis was used to investigate the biological function of dysregulated miRNAs induced by chronic stress in cancer metastasis. Then, we selected miR-337-3p according to sequencing profiles and biological informatics analysis for further study. Finally, we discovered that inhibited expression of miR-337-3p and its downstream target STAT3 might regulate EMT and promote metastasis in breast cancer models suffering chronic stress.

## Results

### Chronic stress promoted breast cancer metastasis in vivo

**Fig. 1:**
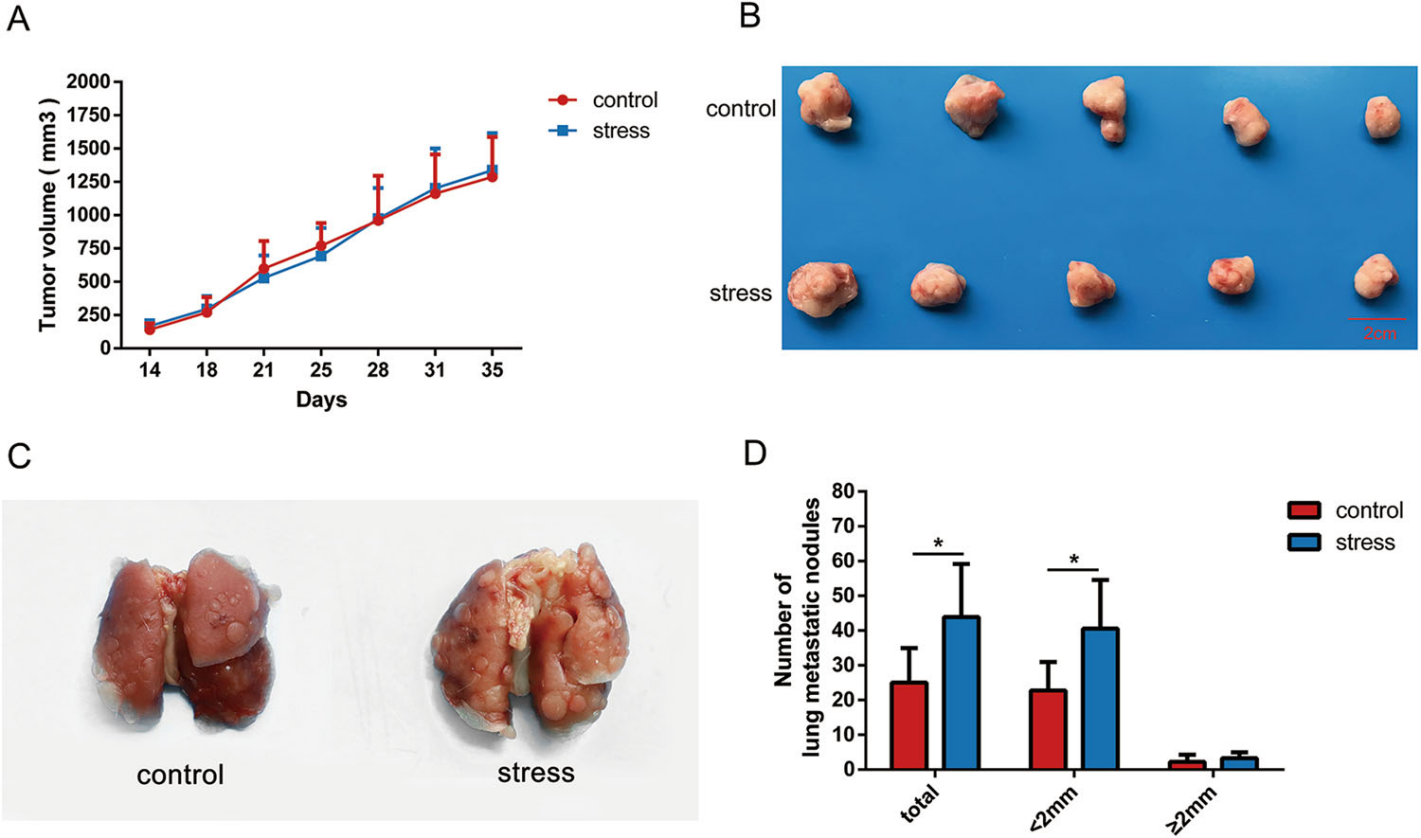
Effect of chronic stress on 4T1 breast cancer in vivo. **a** Quantification of 4T1 primary tumor size in mice of control group and stress group over time (*n* = 5). **b** Images of the tumor xenografts from the control group and stress group are displayed 35 days after cell inoculation. **c** Representative lung images from the control group and stress group. **d** The histogram was used to compare the average numbers of metastatic nodules in the lungs from the control group and the stress group (*n* = 5). Data are shown as the mean ± SD; *P < 0.05.

In order to evaluate the contribution of chronic stress to breast tumor growth and metastasis, 1 × 10^5^ 4T1 cells were subcutaneously inoculated into the right breast region of 6-weeks-old female BALB/c mice to form xenograft model. We observed no significant difference in tumor growth curve and tumor volume between stress group and control group (Fig. 1a, b). However, pulmonary metastasis was more detectable in the mice of stress group (Fig. 1c). Notably, we found that chronic restraint stress increased the number of lung metastatic nodules, especially for those with width less than 2 mm (Fig. 1d). To sum up, we verified that chronic restraint stress contributed to lung metastasis of breast cancer without promoting its growth.

### Chronic stress and norepinephrine (NE) induced EMT in breast cancer in vivo and vitro

**Fig. 2:**
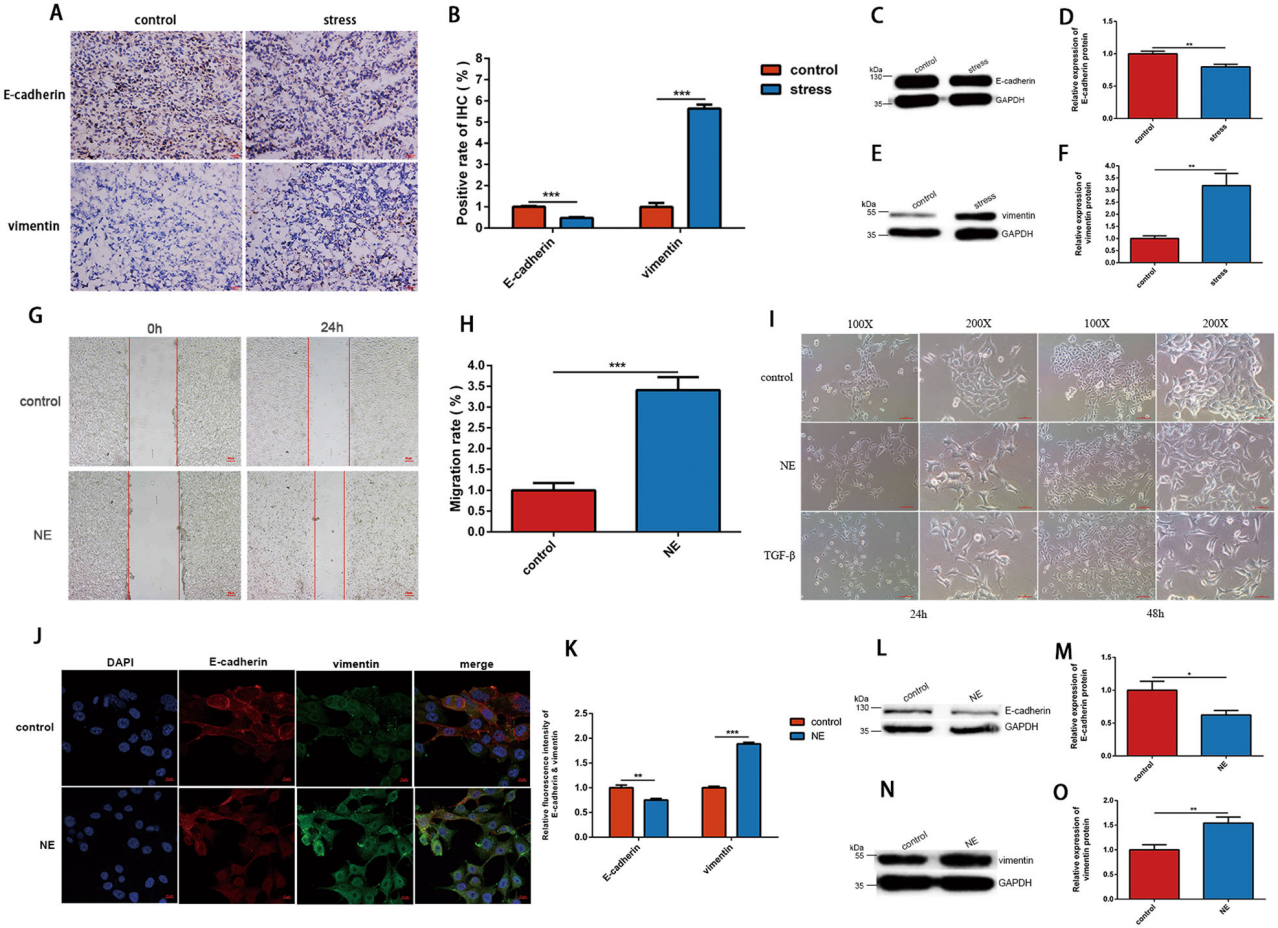
Chronic stress and NE induced EMT of 4T1 cells in vivo and in vitro. **a**, **b** Representative images of immunohistochemical staining of E-cadherin and vimentin protein in tumors from control and stress group (*n* = 3). The fluorescence intensity was quantified and shown in histogram. **c**–**f** Western blot was used to analyze the expression level of E-cadherin and vimentin protein in tumors from control and stress group (*n* = 3). The intensity of the bands for E-cadherin and vimentin was normalized to GAPDH and shown in histograms. **g**, **h** 4T1 cells treated with 10 μM NE showed a significantly accelerated migration speed compared with the control cells treated with normal saline (NS) in wound healing assay (*n* = 3). The migration rate of the two groups are shown in histogram. **i** 4T1 cells were treated with NS, 10 μM NE or 5 ng/ml TGF-β and changes in cell morphology were observed under light microscopy (*n* = 3). Representative optical fields form different groups are shown. **j**, **k** 4T1 cells were treated with NS or 10 μM NE for 48 h, and the EMT-related protein were detected by immunofluorescent staining (*n* = 3). E-cadherin and vimentin are shown in red and green, respectively, while cell nucleus is shown in blue. The fluorescence intensity was quantified and shown in histogram. **l**–**o** 4T1 cells were cultured with NS or 10 μM NE for 48 h. The expression level of E-cadherin and vimentin protein were then detected by western blot (*n* = 3). The intensity of the bands for E-cadherin and vimentin was normalized to GAPDH and shown in histograms. Data are shown as the mean ± SD; *P < 0.05; **P < 0.01; ***P < 0.001.

To determine whether chronic stress contributed to the invasive phenotype of the cancer cells by inducing EMT, we resorted to immunohistochemical staining and western blot analysis to determine the altered expression of EMT-related markers in tumor tissue removed from the mice of two groups above. The results of immunohistochemical staining indicated downregulation of E-cadherin and upregulation of vimentin in stress group compared with control group (Fig. 2a, b), which confirmed the development of EMT in 4T1 breast cancer cells. Furthermore, the western blot analysis demonstrated similar results (Fig. 2c–f).

As one of the most important stress-related hormones, NE was widely used to mimic the effect of chronic stress in vitro experiment. In wound healing assay, after culturing with 10 μM NE, 4T1 cells showed faster migratory speed in comparison with the cells in control group (Fig. 2g, h). Furthermore, we observed that cells incubated with NE became more slender and separated, which was consist with the characteristics of mesenchymal cells induced by TGF-β, while cells in control group showed more epithelial cells features under the light microscope (Fig. 2i). Moreover, immunofluorescence staining demonstrated that expression level of E-cadherin was downregulated and the expression level of vimentin was upregulated in cell cultured with NE (Fig. 2j, k) in vitro and similar observations were obtained in western blot analysis (Fig. 2l–o). Taken together, these results showed that chronic stress and NE induced EMT of 4T1 breast cancer in vivo and in vitro.

### miR-337-3p was downregulated by chronic stress and mediated EMT in 4T1 cells

**Fig. 3:**
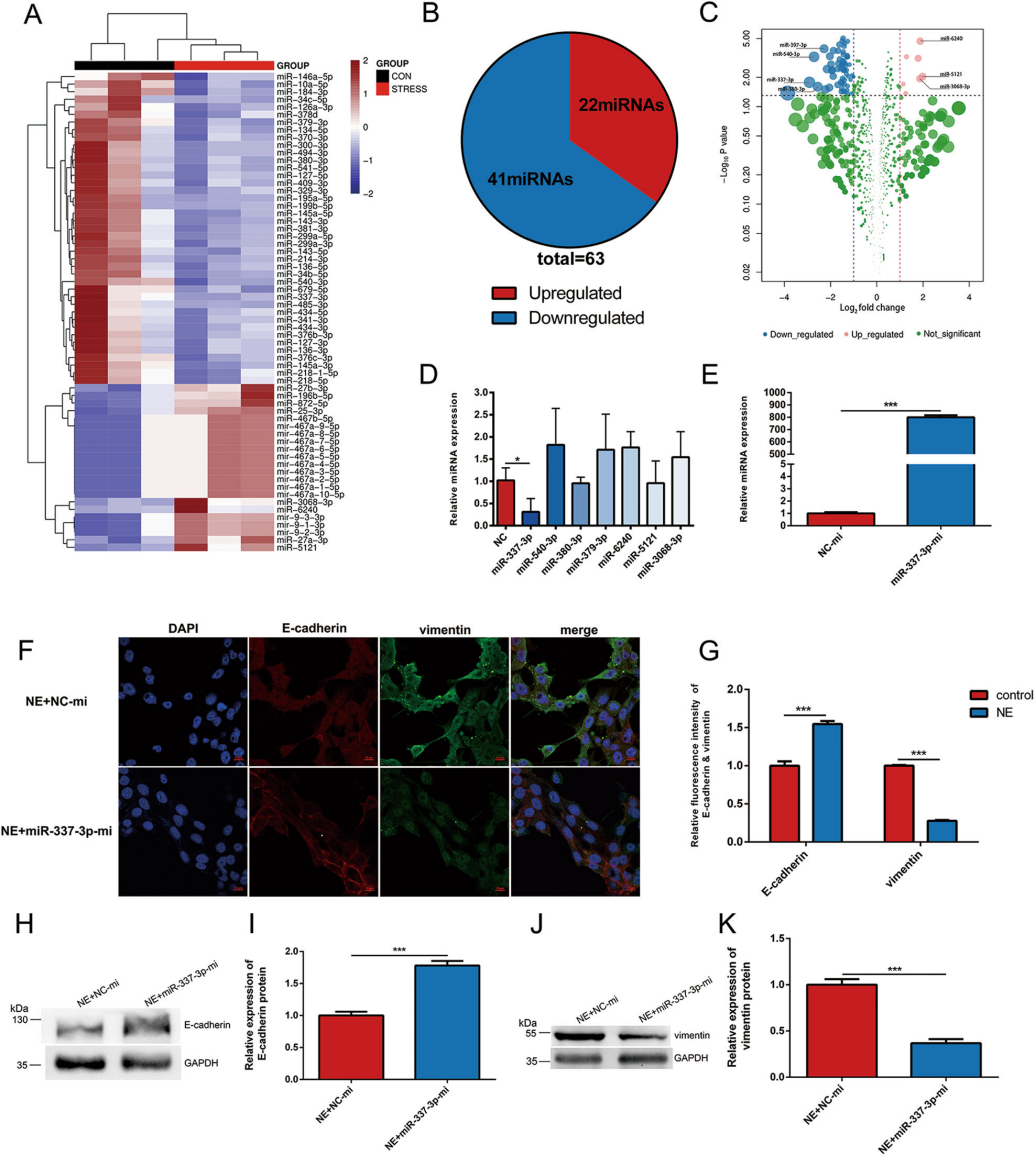
miR-337-3p prevented EMT of 4T1 cells induced by NE. **a** Heat map showing significantly changed (*P* < 0.05, two-tailed Student’s *t*-test) miRNAs between the control group and stress group. **b** Pie chart showing the number of upregulated and downregulated genes in Fig. 3a. **c** Volcano plots showing the specific seven miRNAs with |lg FC| > 1 and *P* < 0.05. **d** RT-qPCR analysis of microRNAs as listed in the figure were carried out in 4T1 cells treated with 10 µM NE for 48 h (*n* = 3). Relative miRNA levels were determined by the ΔΔ Ct method using U6 for internal cross-normalization. **e** The transfection efficacy of the overexpression by miR-337-3p mimics in 4T1 cells was confirmed by RT-qPCR analysis (*n* = 3). Relative miRNA levels were determined by the ΔΔCt method using U6 for internal cross-normalization. **f**, **g** miR-337-3p-mi or NC-mi were transfected into 4T1 cells cultured with 10 µM NE. After 48 h, the EMT related protein were detected by immunofluorescent staining (*n* = 3). E-cadherin and vimentin are shown in red and green, respectively, while cell nucleus is shown in blue. The fluorescence intensity was quantified and shown in histogram. **h**–**k** miR-337-3p-mi or NC-mi were transfected into 4T1 cells cultured with 10 µM NE. After 48 h, the expression level of E-cadherin and vimentin were detected by western blot (*n* = 3). The intensity of the bands for E-cadherin and vimentin was normalized to GAPDH and shown in histograms. Data are shown as the mean ± SD; *P < 0.05; ***P < 0.001.

To figure out the critical role of miRNA in promoting the lung metastasis of breast cancer, we performed miRNA-sequencing on tumor tissues of mice in stress group and control group followed by differentially expressed (DE) analysis. By comparing differentially expressed genes between tumors in two groups, 22 aberrantly upregulated and 41 downregulated candidate genes (*P* < 0.05) were identified in stress group (Fig. 3a, b). Among these, 7 miRNAs (miR-397-3p, miR-540-3p, miR-337-3p, miR-380-3p, miR-6240, miR-5121, and miR-3068-3p) with |lg FC| > 1 were selected (Fig. 3c). The RT-qPCR analysis on miRNAs of 4T1 cells incubated with NE defined miR-337-3p as a possible factor in mediating the chronic stress associated metastasis (Fig. 3d).

To determine the effect of miR-337-3p on NE regulated EMT progression, miR-337-3p mimics (miR-337-3p-mi) was transfected into cultured 4T1 cells to overexpress endogenous miR-337-3p, and transfection efficiency was detected by using RT-qPCR analysis (Fig. 3e). Furthermore, miR-337-3p-mi or negative control mimic (NC-mi) was transfected into 4T1 cells cultured with NE. Immunofluorescence and western blot analysis displayed that both the decreased expression level of E-cadherin and elevated expression level of vimentin induced by NE were suppressed in cells transfected with miR-337-3p-mi (Fig. 3f–k). Taken together, aforementioned information validated that inhibited expression of miR-337-3p might be responsible for acquisition of EMT and cell migration in 4T1 cells cultured with NE.

### miR-337-3p mediated EMT in 4T1 cells by directly targeting STAT3

**Fig. 4:**
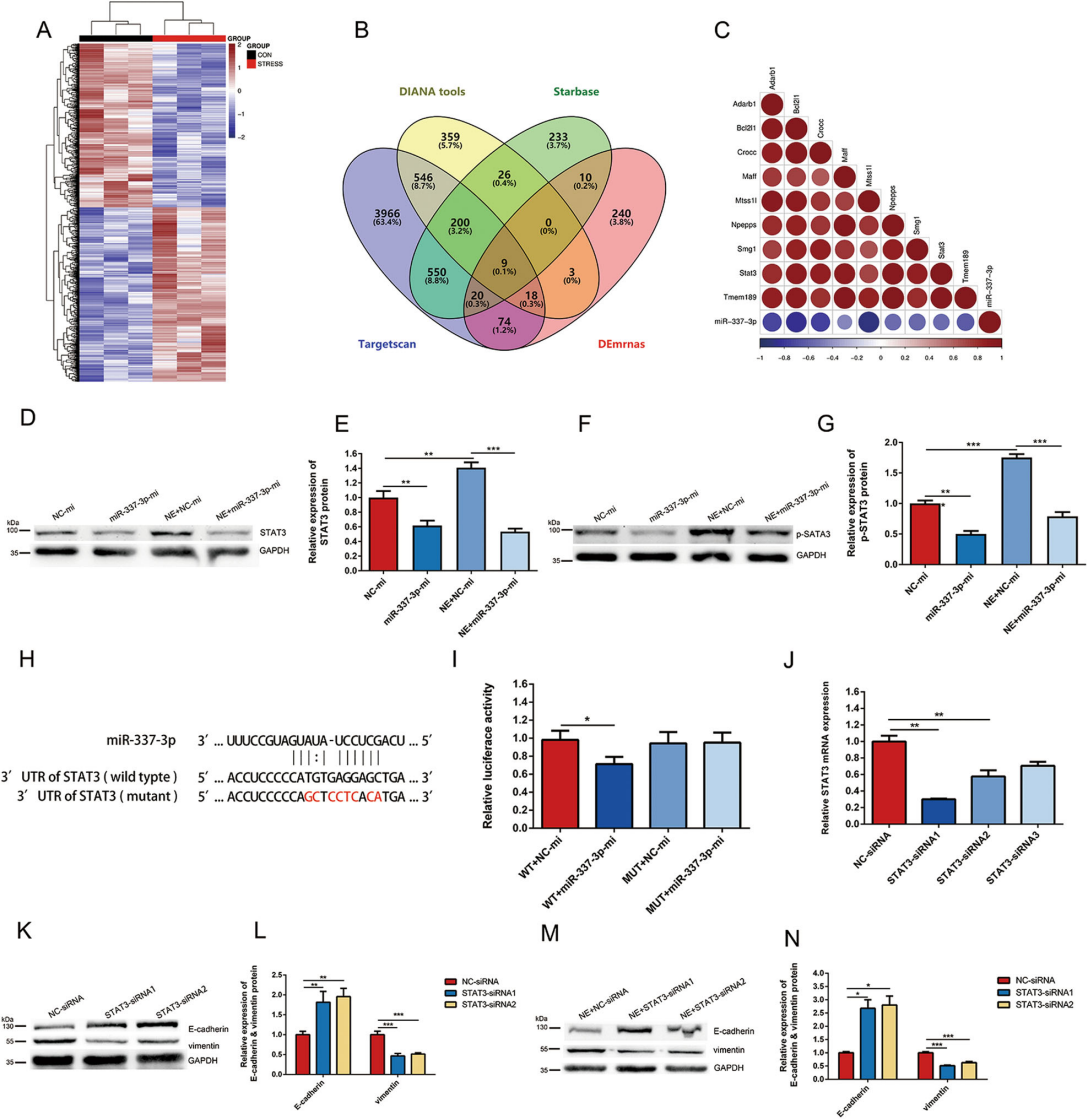
miR-337-3p inhibited EMT of 4T1 cells by suppressing STAT3. **a** Heat map showing significantly changed (*P* < 0.05, two-tailed Student’s *t*-test) mRNAs between control group and stress group. **b** A venn diagram between the differentially expressed mRNAs in **a** and miR-337-3p target genes from three databases (DIANA tools, Starbase, and Target scan) revealed nine potential targets. **c** Correlation heat map showing relationship between the 9 mRNAs in Fig. 4b and miR-337-3p. **d**–**g** miR-337-3p-mi or NC-mi were transfected into 4T1 cells cultured with or without NE. After 48 h, the expression levels of STAT3 and p-STAT3 were detected by western blot (*n* = 3). The intensity of the bands for STAT3 and p-STAT3 was normalized to GAPDH and shown in histogram. **h** The structure and sequence of the miRNA: target interactions for miR-337-3p and the 3′UTRs of STAT3, and the mutant sites in the 3′UTRs of STAT3 is highlighted in red. **i** Luciferase activity assays was performed to confirm the direct binding efficiency of miR-337-3p and its putative target STAT3 (*n* = 3). **j** The transfection efficiency of the knockdown of STAT3 by siRNAs in 4T1 cells was detected by RT-qPCR analysis (*n* = 3). Relative mRNA levels were determined by the ΔΔCt method using GAPDH for internal cross-normalization. **k**–**n** 4T1 cells were transfected with 800 ng/ml STAT3-siRNA1, STAT3-siRNA2, or NC-siRNA and the expression of E-cadherin and vimentin were detected by western blot (*n* = 3). The intensity of the bands for E-cadherin and vimentin was normalized to GAPDH and shown in diagram. Data are shown as the mean ± SD; *P < 0.05; **P < 0.01; ***P < 0.001.

To identify the target mRNA of miR-337-3p involved in EMT progress of 4T1 cells, we also performed mRNA-sequencing on the same tumor tissues from stress group and control group followed by DE analysis. By comparing 374 upregulated genes (*P* < 0.05) in stress group with the mRNAs related to miR-337-3p predicted in three data bases (DIANA tools, Starbase and Target scan), nine genes were screened out (Fig. 4a, b). Moreover, correlation heat map indicated that the nine genes were highly relevant to miR-337-3p (Fig. 4c) and STAT3 was selected for further study as previous evidence proved that it was targeted by miR-337-3p^29,30^, and played a crucial role in EMT^31^.

To investigate whether STAT3 was regulated by NE/miR-337-3p, we used western blot to explore the effect of NE and miR-337-3p on the expression of STAT3 and Phospho-STAT3-Tyr705 (p-STAT3) in 4T1 cells. The results showed that miR-337-3p overexpression by mimics inhibited STAT3 expression as well as p-STAT3 compared with that of group transfected with NC-mi (Fig. 4d–g). We also found that, as expected, NE upregulated STAT3 and p-STAT3 levels, but this effect was suppressed in the cells overexpressing miR-337-3p (Fig. 4d–g). These results demonstrated that STAT3 was a downstream molecule of NE/miR-337-3p signaling pathway.

To determine if STAT3 was directly regulated by miR-337-3p, we used TargetScan to predict the conserved miR-337-3p targeting sites located at 3′-UTR of STAT3 mRNA (Fig. 4h). The luciferase reporter gene assay indicated that after co-transfecting with miR-337-3p mimic and STAT3-WT, the luciferase activity of 4T1 cells was remarkably decreased in contrast to the cells co-transfected with the negative control mimic and STAT3-WT. However, luciferase activity of STAT3-Mut co-transfection system showed no significant difference (Fig. 4i). The above results revealed that STAT3 gene was directly targeted by miR-337-3p.

To evaluate STAT3 mediated the effect of NE on EMT in 4T1 cells, siRNAs were used to knocked down the gene. We applied three different STAT3 siRNAs and selected STAT3–siRNA1 and STAT3–siRNA2 for following experiments based on their better ability to inhibit the STAT3 mRNA (Fig. 4j). In contrast to control group, the increased expression of E-cadherin and decreased expression of vimentin in STAT3–siRNA1 and STAT3–siRNA2 group implied the reverse process of EMT (Fig. 4k, l). Besides, STAT3–siRNA1 and STAT3–siRNA2 had an effect on reversing the EMT progression induced by NE (Fig. 4m, n). The results above confirmed that STAT3 was a significant molecule negatively regulated by miR-337-3p and contributed to the progression of EMT.

### miR-337-3p inhibited lung metastasis and EMT of 4T1 tumor cells in vivo

**Fig. 5:**
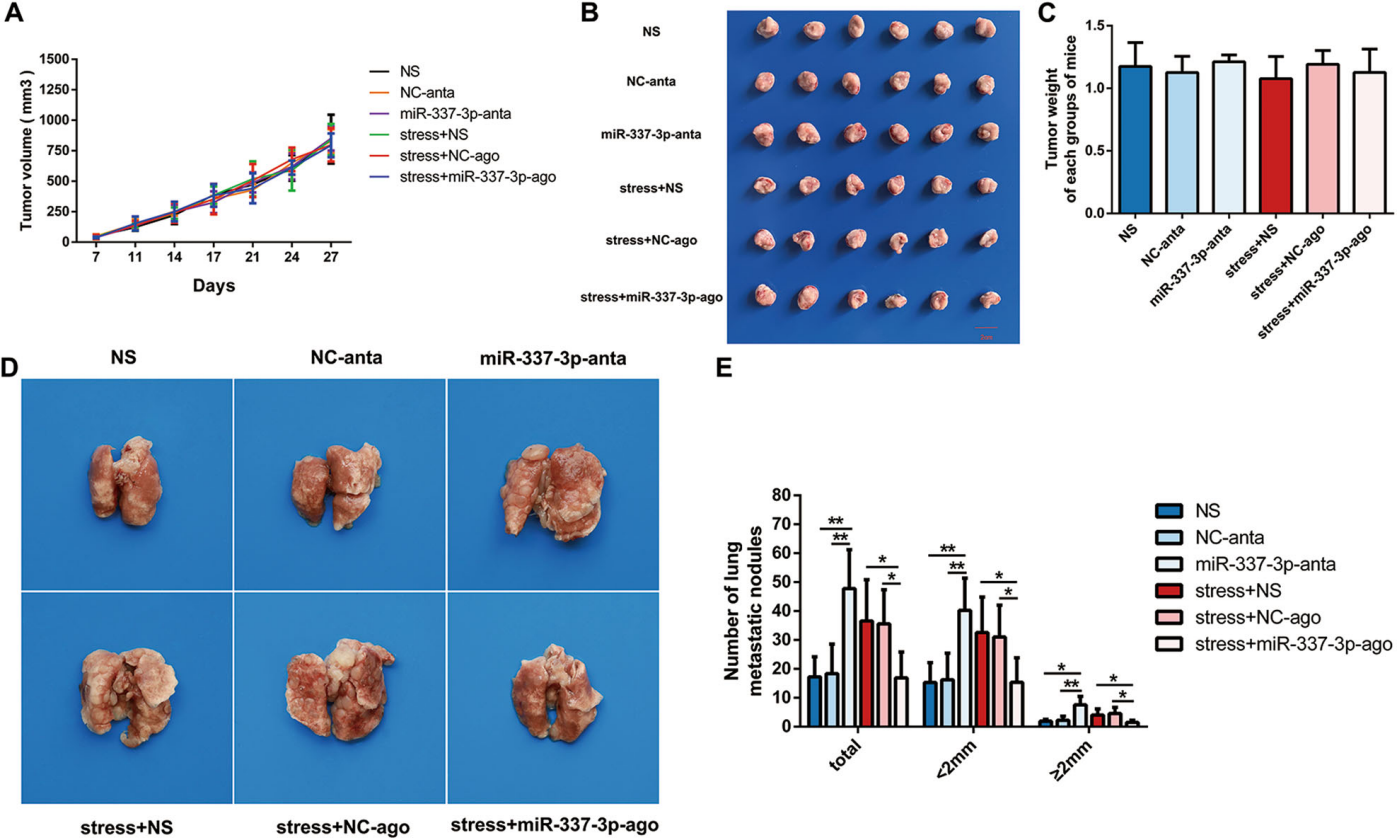
miR-337-3p inhibited lung metastasis of 4T1 breast cancer. **a** The volume of the tumor during the process of tumor growth at seven different time points was determined (*n* = 6). **b** The tumor diameters of the six groups were measured 31 days after cell inoculation (*n* = 6). (1) NS group, injected with normal saline intratumorally; (2) NC-anta group, injected with negative control for antagomir intratumorally; (3) miR-337-3p-anta group, injected with miR-337-3p antagomir intratumorally; (4) stress + NS group; being restrained and injected with normal saline intratumorally; (5) stress + NC-ago group; being restrained and injected with negative control for agomir intratumorally; (6) stress + miR-337-3p-ago group, being restrained and injected with miR-337-3p agomir. **c** The tumor weight of the six groups is shown in diagram (*n* = 6). **d** Representative lung images from the six groups are displayed 31 days after cell inoculation. **e** The number of metastatic nodules of lungs from tumor-bearing BALB/c mice in six groups were calculated (*n* = 6). Data are shown as the mean ± SD; *P < 0.05; **P < 0.01.

To explore the effect of miR-337-3p downregulation on tumor growth and metastasis in vivo, antagomir was injected intratumorally in 4T1 tumor bearing mice for silencing the miR-337-3p. We observed that the tumor growth curves of each group were similar (Fig. 5a). Furthermore, there was no significant difference in the size and weight (Fig. 5b, c) of the tumors removed from each group. However, we found that the number of lung metastases in miR-337-3p-anta group (injected with miR-337-3p antagomir intratumorally) was significantly increased compared to that in NS group (injected with normal saline intratumorally) and NC-anta group (injected with negative control for antagomir intratumorally) (Fig. 5d, e). Furthermore, agomir was injected intratumorally to overexpress miR-337-3p in 4T1 tumor bearing mice under chronic restraint stress. We found that there was no significant difference in the tumor growth curve, tumor volume and tumor weight in each group (Fig. 5a–c), while the number of lung metastases in stress + miR-337-3p-ago group (being restrained and injected with miR-337-3p agomir intratumorally) decreased compared to that in stress + NS group (being restrained and injected with normal saline intratumorally) and stress + NC-ago group (being restrained and injected with negative control for agomir intratumorally) (Fig. 5d, e). These results indicated that miR-337-3p could inhibit chronic stress associated lung metastasis without influencing the growth of 4T1 xenograft.

**Fig. 6:**
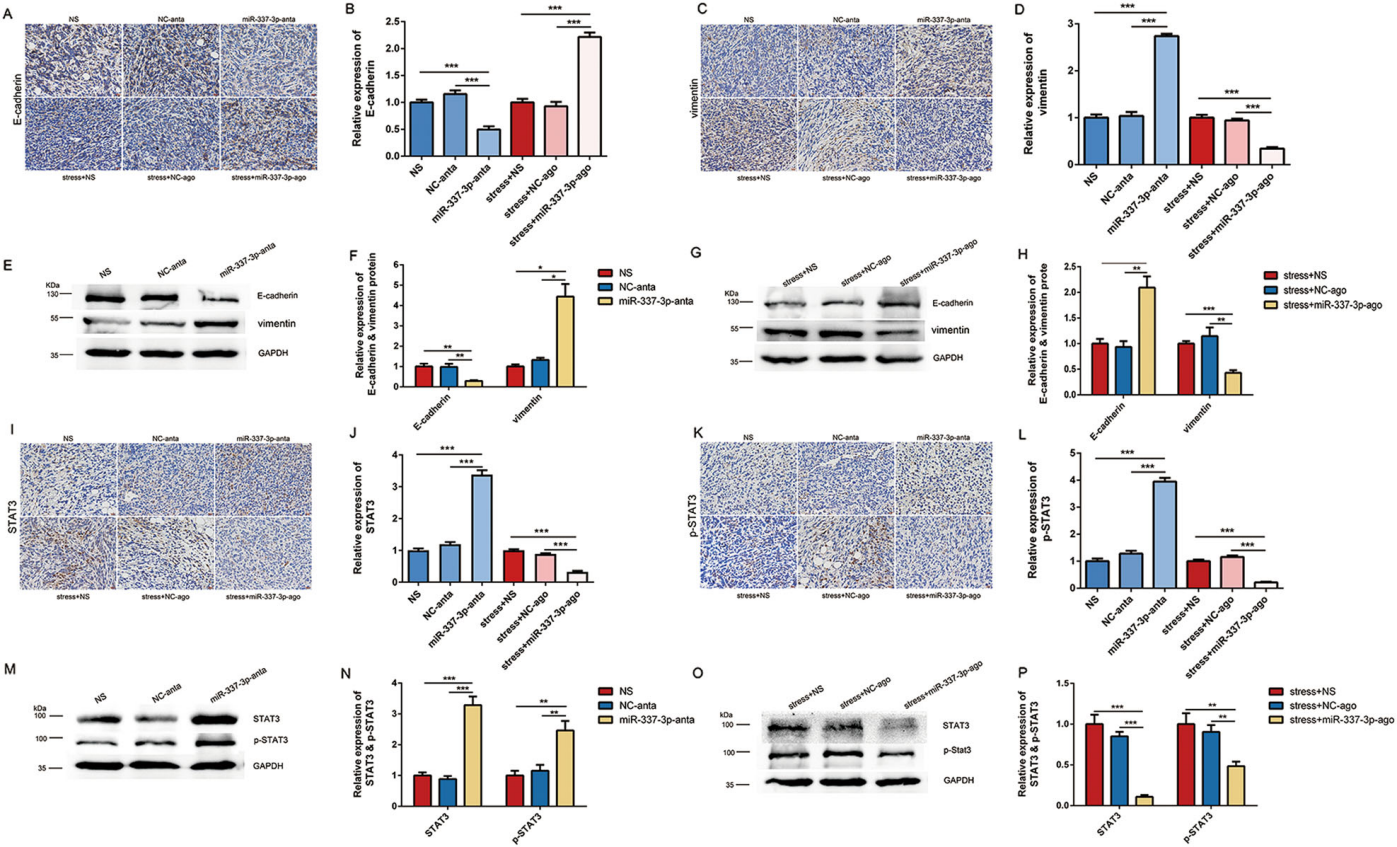
miR-337-3p inhibited EMT of 4T1 cells in vivo. **a**–**d** Immunohistochemical staining of E-cadherin and vimentin in tumor samples of six groups (*n* = 3). **e**–**h** The expression of E-cadherin and vimentin in tumor samples of six groups were detected by western blot (n = 3). The intensity of the bands was normalized to GAPDH and shown in histogram. **i**–**l** Immunohistochemical staining of STAT3 and p-STAT3 in tumor samples of six groups (*n* = 3). **m**–**p** The expression of STAT3 and p-STAT3 in tumor samples of six groups were detected by western blot (*n* = 3). The intensity of the bands was normalized to GAPDH and shown in histogram. Data are shown as the mean ± SD; *P < 0.05; **P < 0.01; ***P < 0.001.

To verify whether miR-337-3p inhibited EMT of 4T1 cancer cells in vivo, immunohistochemical staining and western blot analysis were used to detect the expression of related proteins. The results of immunohistochemical staining showed that silencing miR-337-3p by antagomir downregulated expression of E-cadherin while upregulated expression of vimentin. In addition, overexpression of miR-337-3p by agomir suppressed downregulation of E-cadherin and upregulation of vimentin induced by chronic restraint stress (Fig. 6a–d). Same results were obtained in western blot analysis (Fig. 6e–h).

**Fig. 7:**
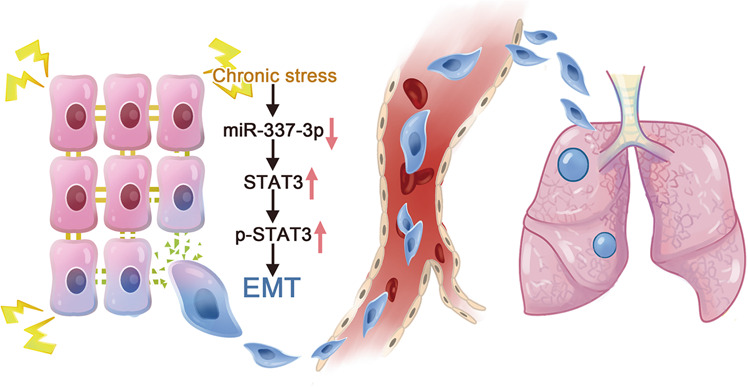
Schematic representation of the proposed mechanism of chronic stress induced EMT and metastasis. Downregulated miR-337-3p induced by chronic stress could futher activate STAT3 and promote EMT progression in breast cancer cells, which might contribute to lung metastasis.

To determine whether miR-337-3p regulated STAT3 and p-STAT3, we resorted to immunohistochemical staining and western blot analysis to detect the expression of the two proteins. The results from immunohistochemical staining indicated that silence of miR-337-3p by antagomir increased STAT3 and p-STAT3 expression. Moreover, the effect of stress on increasing STAT3 and p-STAT3 expression was suppressed by overexpression of miR-337-3p (Fig. 6i–l). Same results were acquired in western blot analysis (Fig. 6m–p). To sum up, the aforementioned results implied the critical role of miR-337-3p in regulating EMT and metastasis in vitro and in vivo, the proposed mechanism of which was shown in the schematic representation (Fig. 7).

## Discussion

Patients with cancer tend to suffer chronic stress triggered by psychological disorders during the course of therapy and follow-up^32^. It has been well-reported that chronic stress could promote the release of stress-related hormone like NE through the activation of sympathetic nervous system or hypothalamic-pituitary-adrenal axis^33^. Prolonged exposure to stress-related hormones contributes to tumor growth, invasive capacity, and angiogenesis in various cancers^10,34–36^. Clinical and epidemiological studies also indicated that stress-related factors are related with cancer progression and poor outcomes^37^. A retrospective research indicated that psychological disorders among prostate cancer patients might be related to increasing invasive capability of tumor and metastasis^38^. In this study, we also found that chronic restraint stress contributed to lung metastasis of 4T1 xenograft rather than tumor growth. Researchers exploring the effect of chronic stress on various cancers acquire contradictory results in the primary tumor growth, which may attribute to different cell lines and experimental design. However, they obtained similar results in chronic stress-related metastasis^39–43^.

Accumulating researches revealed the important role of EMT in the invasive capability and mobility of cancer cells^44^. Activation of the EMT triggers a cascade of biological events to induce morphological changes in epithelial cells and bestows them metastatic phenotype, such as loss of cell junction and gain of mobility^45^. E-cadherin is a crucial protein participating in the cellular adhesions of epithelial cells and suppresses the metastasis of epithelial carcinomas^46^. Vimentin acts as a key factor in the final stage of EMT program and indicates the highly proliferative and invasive state of mesenchymal-like carcinoma cells^47^. Consistent with previous studies^48,49^, our study demonstrated that chronic stress and its related hormone NE downregulated the E-cadherin expression and upregulated the vimentin expression in vivo and in vitro. Additionally, as shown in Fig. 2, 4T1 cells cultured with NE became more elongated and separated compared to the control group and showed increased migration capacity. In all, the changes in molecular phenotype, cellular morphology and migration capabilities supported the development of EMT.

Previous studies validated that various cytokines, signaling pathway, and the interaction between tumor cells and extracellular matrix components all involved in the regulation of EMT^50,51^. Our previous work indicated two underlying signaling transduction pathways, namely β-AR/TGF-β1/p-Smad3/Snail and β-AR/TGF-β1/HIF-1α/Snail, in the NE regulated EMT program in HT-29 and A549 cell lines^52^. In addition, NE could induce expression of human telomerase reverse transcriptase (hTERT) to promote ovarian cancer cell EMT and invasion by regulating ADBR2/PAK/Src/HIF-1α and c-Myc signaling pathway^53^. In recent years, miRNAs are proved to be closely connected with the progression of EMT^54^. However, no research has confirmed the contributions of miRNA to the EMT induced by NE so far. In our study, next-generation sequencing and bioinformatic analysis were used to screen out differentially expressed miRNAs induced by chronic stress. Seven miRNAs were identified based on the criteria of *P* < 0.05 and | log^2^ (fold change) | (|lg FC|) > 1. Among which, we selected miR-337-3p for further investigation, as its expression in 4T1 cells cultured with NE detected by RT-qPCR analysis was in consistent with the trend in bioinformatic prediction. The function of miR-337-3p in tumor progression has been illustrated by a few researchers. miR-337-3p acts as a suppressive role to modulate cell proliferation, EMT, and metastasis by targeting Capn4 in clear cell renal cell carcinoma^19^. Additionally, miR-337-3p suppresses proliferation, migration, and invasion of cervical cancer cells by targeting Rap1A and the expression levels of miR-337-3p is related to better clinicopathological characteristics of cervical cancer^55^. We also found that overexpression of miR-337-3p by mimics reversed the NE induced EMT in 4T1 cells. Besides, miR-337-3p acted as a suppressor of EMT program in vivo. Taken together, miR-337-3p was an underlying regulator of chronic stress-related metastasis.

As a transcription factor in cytoplasmic, STAT3 could be activated in the cell signaling cascade in various cancers and acts as an important component in both EMT process and metastasis^56–58^. In our study, the results of bioinformatics analysis suggested that STAT3 might be a possible target gene of miR-337-3p. Furthermore, expression levels of STAT3 and p-STAT3 were upregulated by NE, and the phenomenon was reversed after overexpressing miR-337-3p in 4T1 cells. In vivo, STAT3 and p-STAT3 were also negatively regulated by miR-337-3p in 4T1 tumor models. We revealed the targeting relationship between miR-337-3p and STAT3 via dual-luciferase reporter gene assay. miR-337-3p could negatively regulate the JAK2/STAT3 signaling cascade to promote cell proliferation and migration of hepatocellular carcinoma^59^. The sensitivity of nonsmall cell lung cancers to taxane also might be attributable to STAT3 targeted by miR-337-3p^60^. The direct targeting relationship between miR-337-3p and STAT3 contributes to the biological process of metastasis. Besides, we found that knockdown of STAT3 by siRNAs led to a reverse of EMT process, and suppressed NE induced EMT. The above results further supported the hypothesis that psychological disorders could promote metastasis.

To sum up, we demonstrated for the first time the involvement of miR-337-3p and its target gene STAT3 in regulating EMT program of breast cancer under chronic stress in vivo and in vitro. Further investigation of the mechanism underlying the metastasis effects of psychological disorders in cancer patients is warranted. The influence of psychological factors on tumor progression of patients requires more attention in clinical.

## Materials and methods

### Cell culture

All cell lines used for experiments were acquired from American Type Culture Collection (Manassas, VA, USA). RPMI-1640 medium (Gibco, Carlsbad, CA, USA) was used for cultivating the 4T1 tumor cells and Dulbecco’s modified Eagle’s medium (Gibco, Carlsbad, CA, USA) was used for incubating the HEK-293T cells, both mediums containing 10% FBS. All cells were placed in an incubator with 5% CO_2_ and 95% saturated humidity at 37 °C, and the medium was changed every two days.

### Mice model

The 6-weeks-old BALB/c female mice were provided by Beijing Huafukang Bioscience (Beijing, China). To induce tumors, 1 × 10^5^ 4T1 cells suspended in 100 μl of saline injected subcutaneously into the right breast region of each mouse.

Mice model 1: Mice model 1 were used for exploring the effect of chronic restraint stress on tumor. In this model, ten mice were randomly divided into control group and stress group 7 days after 4T1 cell inoculation. Mice in stress group were restrained in drafty 50 ml conical bottom centrifuge tubes 2 h daily for consecutive 28 days, while mice in control group were free to move. The theory foundation of chronic restraint stress model were detailedly elucidated in our previous work^61^.

Mice model 2: Mice model 2 were used for verifying the effect of miR-337-3p on metastasis and EMT in vivo. In this model, 36 mice were randomly divided into six groups according to different treatments 7 days after 4T1 cell inoculation: (1) NS group, injected with normal saline intratumorally; (2) NC-anta group, injected with negative control for antagomir (Ribobio, Guangzhou, China) intratumorally; (3) miR-337-3p-anta group, injected with miR-337-3p antagomir (Ribobio, Guangzhou, China) intratumorally; (4) stress + NS group; being restrained and injected with normal saline intratumorally; (5) stress + NC-ago group; being restrained and injected with negative control for agomir (Ribobio, Guangzhou, China) intratumorally; (6) stress + miR-337-3p-ago group, being restrained and injected with miR-337-3p agomir (Ribobio, Guangzhou, China) intratumorally. The mice were restrained 2 h daily and the intratumor injection was administrated twice a week for 7 times in total. Tumors were measured using vernier caliper twice a week, and volume was calculated using the method described by Seiji Naito^62^. In order to count metastases, lungs were soaked in saline solution and cleaned. Photographs of the tumors and lungs were taken and the number of nodules on the surface of lungs was quantified. All animal experiments were carried out in conformity with the Guide of the Animal Care and Use Committee of West China Hospital, Sichuan University, China.

### Specimens and sequencing

For the next-generation sequencing on Illumina Hiseq 2500/2000 platform, miRNAs and mRNAs were extracted from tumor tissues of control group and stress group in mice model 1. After removing adapter sequences, collapsing reads with the same sequence, and reading with less than 15 bases in length from raw data, clean data were obtained and analyzed based on the miRDeep2(2.0.0.8). miRNA and mRNA reads were mapped to mice miRNA precursors from miRBASE release 22.1 using miRDeep2 allowing zero mismatches. Mapped reads were then quantified by miRDeep2. Analysis of the differentially expressed miRNAs and mRNAs between the control group and stress group were performed by the DEseq2 R package. The significance threshold was set as *P* < 0.05 & | log^2^ (fold change) | >1 in this research.

### miRNA, siRNA and plasmid transfections

5 × 10^4^ cells/well 4T1 cells were plated onto 12-well plate for 24 h in a humidified incubator of 5% CO_2_ and 37 °C. The transfection of all miRNA mimics and siRNAs were performed using Lipofectamine™ RNAiMAX Transfection Reagent (Invitrogen, Carlsbad, California, USA) in guidance with the standard protocol, and reporter gene plasmid was transfected through the use of Lipofectamine 3000 reagent (Invitrogen, Carlsbad, California, USA). The cells were collected for next experiments after 6 h of transfection.

### Wound healing assay

5 × 10^5^ cells/well 4T1 cells were planted into 6-well plates for incubation at 37 °C. We used a sterilized tip of transfer liquid gun to gently draw lines on the plate when cells covered the bottom of the plate, and the width of all scratches were required to be about the same. After removing cell culture fluid and washing the plate with PBS buffer for three times to wash away cell debris produced by scratches, cells were photographed (0 h). Next, serum-free medium was added into the plate for blocking cell proliferation and the cells were photographed at the same points again after incubation at 37 °C for 24 h. The OLYMPUS inverted microscope (Tokyo, Japan) was used to take photos the scratches and the area of cells migrating to the scratched area was estimated by using Image J software.

### Immunohistochemistry

After being incubated at 65 °C for 30 min, the sections were subjected to deparaffination by using xylene, transferred to gradient ethyl alcohol (100% ethanol, 5 min; 95% ethanol, 2 min; 90% ethanol, 2 min; 80% ethanol, 2 min), washed with PBS for 2 min. Then, the sections were boiled with 0.01 M citric acid buffer (pH 6.0) in a heated bath of 97 °C for 40 min to repair antigen, soaked in 3% H_2_O_2_ for 30 min for quenching the exogenous peroxidase. After blocking with goat serum for 30 min, the sections were exposed with the following primary antibodies, rabbit antimouse antibody to E-cadherin (Proteintech, Wuhan, China) and chicken antimouse antibody to vimentin (Abcam, Cambridge, UK) at 37 °C for 2 h and washed with PBS. Subsequent incubation was performed with the secondary antibodies labeled with horseradish peroxidase, goat antichicken antibody to immunoglobulin Y (IgY) (Abcam, Cambridge, UK) and goat antirabbit antibody to immunoglobulin G (IgG) (Jackson, Philadelphia, Pennsylvania, USA), at 37 °C for 30 min. The sections were then counterstained with hematoxylin (Bioss, Beijing, China). After sealing with Clear-Mount (Head biotechnology, Beijing, China), the slides were observed under an electron microscope (Zeiss, Oberkochen, Germany) with images subsequently obtained.

### Immunofluorescence

2 × 10^4^ cells/well 4T1 cells were inoculated in a 24-well culture plate with cell slides placed at the bottom in advance, so that the cells expand to 60–70% before experiment. The cells on the slides were washed by PBS for three times and then fixed with 500 μl 4% polyformaldehyde at room temperature for 15 min. After that, pre-cooled 0.2% Triton X-100 diluted in PBS was used to permeabilize the cells for 5 min. Then, the cells in each well were blocked with 500 μl goat serum containing 0.3% Triton X-100 at room temperature for an hour. After removal of the goat serum, the cells were covered with the mixed primary antibodies of E-cadherin (CST, Boston, Massachusetts, USA) and vimentin (Abcam, Cambridge, UK) overnight at 4 °C under dark conditions, and the two primary antibodies were coupled with Alexa Fluor® 594 and Alexa Fluor® 488, respectively. Afterwards, cells were washed three times using PBS, stained with DAPI staining solution (Invitrogen, Camarillo, CA, USA) for 10 min, washed, and sealed with BrightMount (Abcam, Cambridge, UK). Cells on the slides were observed by using a fluorescence confocal microscope (Zeiss, Oberkochen, Germany) with images subsequently obtained.

### Real-time quantitative polymerase chain reaction (RT-qPCR) analysis

miRNA was extracted with a miRcute miRNA extraction and separation kit (TIANGEN, Beijing, China) while total RNA was extracted with Trizol (Takara, Shiga, Japan). After measuring the concentration, miRNA and mRNA was reversely transcribed into cDNA with a miRcute Plus miRNA First-Strand cDNA Kit (TIANGEN, Beijing, China) and PrimeScript RT reagent Kit with gDNA Eraser (Takara, Shiga, Japan), respectively. Expression analysis of miRNA and mRNA was performed on a LightCycler 96 System (Roche, Basel, Switzerland) by using miRcute Plus miRNA qPCR Kit (TIANGEN, Beijing, China) and TB Green® Premix Ex Taq™ II (Takara, Shiga, Japan), respectively. All primers for miRNA were ordered form GeneCopoeia Inc. (Guangzhou, China) and primers for mRNA were synthesized by GenePharma Co. (Shanghai, China), the sequences of all primers are shown in Table S1. U6 and GAPDH were used as the internal control for miRNAs and mRNA, respectively. The Ct value was recorded and U6 was served as the internal control for miRNA, while glyceraldehyde-3-phosphate dehydrogenase (GAPDH) serving as the internal control for STAT3. The ΔΔ Ct method was used for calculating the relative expression of genes.

### Western blots

The total protein from tumor tissues or cells was extracted with RIPA lysis buffer (Beyotime, Shanghai, China) containing inhibitors of protease and phosphatase. The concentration of each protein sample was detected by using BCA protein assay kit (Solarbio, Beijing, China) in line with the manufacturer’s recommendation. For electrophoresis, same quantity of protein lysate was added to each lane and then transferred onto the Polyvinylidene fluoride (PVDF) membrane (Bio-Rad, California, USA). Next, the membrane was soaked into the 5% defatted milk for 1 h in order to block the non-specific sites. Then, the membrane was covered overnight at 4 °C with the following primary antibodies: rabbit antimouse antibodies against E-cadherin (Proteintech, Wuhan, China), vimentin (Bioss, Beijing, China), STAT3 (Abcam, Cambridge, UK), p-STAT3 (Abcam, Cambridge, UK) and mouse antimouse antibody GAPDH (ZenBio, Chengdu, China). After washing five times by Tris-Buffered Saline with Tween-20 (TBST), the membranes were immersed into TBST with the secondary antibody, HRP-labeled goat antirabbit IgG (ZenBio, Chengdu, China) and goat antimouse IgG (ZenBio, Chengdu, China), respectively, for 1 h. After washing with TBST for three times, the membrane was treated with the Immobilon western HRP Substrate (Millipore, Bedford, MA, USA) and the bands were detected by iBright CL1000 Imaging System (Invitrogen, Carlsbad, California, USA). The relative intensity of each blot was assessed and analyzed by using the Image J Software.

### Dual-luciferase reporter gene assay

The pEZX-FR02-WT-STAT3 (STAT3-WT) containing the 3′-UTR region and pEZX-FR02-Mut-STAT3 (STAT3-Mut) containing the mutated binding site of miR-337-3p were constructed (GeneCopoeia, Guangzhou, China). In guidance with protocol of previous publication^63,64^, NC mimic and miR-337-3p mimic were co-transfected with STAT3-WT or STAT3-Mut into HEK-293T cells. After 48 h of incubation, cells were subjected to measure the luciferase activity on the basis of specification of the dual-luciferase reporter gene assay kit (Beyotime, Shanghai, China). The fluorescence intensity was detected using SpectraMAX i3x Ptatform (Molecular Devices, California, USA).

### Statistical analysis

The statistical analyses of all experimental data were carried out using GraphPad Prism software version 6.0. (GraphPad Software, Inc., California, USA). All measurement data were presented in a form of mean ± SD. Student’s *t*-test (two-sided) analyses were performed between each two groups. *P* < 0.05 was regarded as an indication of significant differences.

## Supplementary information

Table S1

Code used for variance analysis
